# Influence of Pore Structure on Compressive Strength of Cement Mortar

**DOI:** 10.1155/2014/247058

**Published:** 2014-03-16

**Authors:** Haitao Zhao, Qi Xiao, Donghui Huang, Shiping Zhang

**Affiliations:** ^1^College of Civil and Transportation Engineering, Hohai University, Nanjing 210098, China; ^2^China Three Gorges Corporation, Beijing 100038, China; ^3^Department of Civil Engineering and Architecture, Nanjing Institute of Technology, Nanjing 211167, China

## Abstract

This paper describes an experimental investigation into the pore structure of cement mortar using mercury porosimeter. Ordinary Portland cement, manufactured sand, and natural sand were used. The porosity of the manufactured sand mortar is higher than that of natural sand at the same mix proportion; on the contrary, the probable pore size and threshold radius of manufactured sand mortar are finer. Besides, the probable pore size and threshold radius increased with increasing water to cement ratio and sand to cement ratio. In addition, the existing models of pore size distribution of cement-based materials have been reviewed and compared with test results in this paper. Finally, the extended Bhattacharjee model was built to examine the relationship between compressive strength and pore structure.

## 1. Introduction 

Natural sand has been traditionally used in mortars and concrete. However, as growing environmental restrictions to the exploitation of sand from riverbeds, an alternative material to produce fine aggregates should be developed. Manufactured fine aggregate then appear as an attractive alternative to natural fine aggregates for cement-based materials. Manufactured sand (MS), in contrast with natural sand (NS), comes from the mechanical crushing of virgin rock, which has angular particle and rough texture. Wakchaure and BE [1] demonstrated that manufactured sand contains more microfines, which provide a larger area of the interfacial bond zone, and angular shape and rough texture improve the frictional properties that increased the flexural strength of concrete. Some other experiments illustrated that concrete with manufactured sand had good abrasion resistance, chloride ion permeability, freeze-thaw durability, and some other durability [2–4]. In a word, these existing researches had indicated that manufactured sand can be used effectively and economically in concretes designed for a variety of applications.

The pore structure of cement-based materials contains air voids, capillary pores, and gel pores, and the pores are randomly sized, arranged, and connected [5]. It is a well-known fact that porosity is one of the key parameters which directly affect the strength and durability of cement-based materials [6, 7]. As we all know, a lower porosity in concretes with sufficient binding material content leads to higher strength concretes [8–12]. According to Mindess et al. [13], the traditional classification of pores in concrete, two classes of pores were considered: gel pores (<10 nm), which are associated with the formation of hydration products, and capillary pores (10 nm~10000 nm), which dominate transport processes. And the dependence of diffusivity of concrete on pore structure attributes mainly to the effect of capillary porosity and connectivity of these capillary pores [14].

To determine the microstructure of cement-based materials, different techniques were developed and each has its own limitations. Chlorides penetration method [15] has no limitation on the size of samples, and surface area and pore structure determined by desorption isotherm demonstrated that the 3D nondestructive micro-CT technique is limited by their resolution [16, 17]. Mercury intrusion porosimetry (MIP) is based on the premise that a nonwetting liquid (contact angle greater than 90°) will only intrude capillaries under pressure according to the Washburn equation. Theories indicated that the MIP technique is an available method to determine the pore size distribution of cement-based materials.

Many pore size distribution models of cement-based materials have been proposed according to MIP experiment results [18–22]. In these models, mean pore diameter, threshold radius, and total porosity are the most crucial parameters describing pore size distribution. Atahan et al. [23] indicated that the mean pore diameter, hydration degree, and threshold radius are the main characteristics of the material which accounts for their permeability properties. Odler and Rößler [6] have prepared cement pastes with different water cement ratios which were hydrated at different temperatures for different ages. They have demonstrated that the main factor influencing strength properties is the porosity. Winslow and Liu [24] investigated the difference of the pore size distribution between the cement paste in concrete and mortar and the plain paste. They showed that there was an additional formation of pores greater in size in mortar and concrete than that of the plain paste, and they considered that these larger pores are present only in the interfacial zone between aggregate and cement paste.

Many researchers have proposed various relationships between strength and porosity of porous materials [8–12]; however, porosity is not the only influential factor on strength. As experimental research indicated, finer pores concrete has higher strength than the coarser one at the same total porosity. So, it is indispensable to research the relationship between the strength and pore structure of cement-based materials [6, 12, 24].

In this work, the MIP technique was used to investigate the effect of water to cement ratio (*w*/*c*) and sand to cement ratio (*s*/*c*) on the pore structure of manufactured sand mortar and natural sand mortar. The existing models of pore size distribution of cement-based materials have been reviewed and compared with test results in this investigation. In addition, uniaxial compressive strength was tested and the strength-pore structure relationship of cement mortar has been constructed in this paper.

## 2. Experimental Programme

### 2.1. Experimental Materials and Mix Proportions

**Figure 1:**
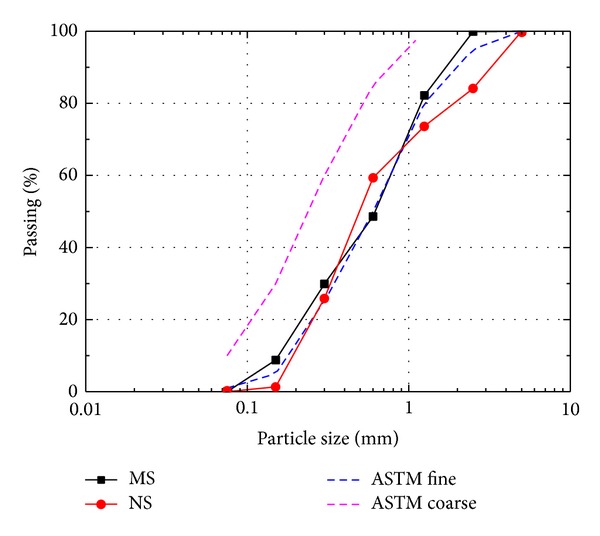
Particle size distribution of fine aggregate according to ASTM C-33.

Ordinary Portland cement (ASTM Type I) without mineral additions was used as binder. In this work, 24 mix proportions cement mortar with the *w*/*c* of 0.4, 0.5, 0.6, and 0.7 were prepared. The fine aggregate was manufactured sand consisting mainly of calcium carbonate and natural river sand composed mostly of quarts. The gradation of the two types of fine aggregate was shown in Figure 1, and the physical characteristic was shown in Table 1. The mix proportion and workability of cement-based mortars are listed in Table 2.

### 2.2. Strength Measurement

Compressive tests were run on specimens according to ASTM C349. The specimens (52 mm × 100 mm cylinder) were prepared by drilling cores. 0.4 kN/s uniform continuous loads were loaded on the specimens using a new SANA hydraulic universal testing machine.

### 2.3. Determination of Porosity

The total porosity of the mortar samples was estimated from the ratio between the weight of the test sample after curing in an oven at 105°C until reaching constant weight, followed by saturation of the sample by immersion in water for 72 hours, according to the ASTM C642-97. This is the fraction of pore volume accessible to water in the mortar. The porosity was calculated using the following equation:

(1) $$\begin{array}{c} p=\frac{\left(W_{\text{ssd}}-W_{d}\right)}{\left(W_{\text{ssd}}-W_{w}\right)}\times 100\%, \end{array}$$

where *p* is the porosity (100%), *W*_ssd_ is the specimen weight in the saturated surface-dry (SSD) condition (*g*), *W*_*d*_ is the specimen dry weight until reaching constant weight in oven (*g*), and *W*_*w*_ is the weight of saturated specimen (*g*).

### 2.4. Mercury Intrusion Porosimetry (MIP)

MIP is a widely used method for measuring the pore size distribution of cement-based materials. In the MIP test, samples are intruded into a chamber; after the chamber is evacuated, the samples are surrounded by mercury, and the pressure ranges from subambient to 60,000 psi (414 MPa). The contact angle and surface tension of mercury were assumed to be 117° and 0.484 N/m, respectively. On the pressure, the smallest pore size into which mercury can be intruded is 2 nm and the largest pore size which can be intruded is 200 mm with subambient pressure [25]. The MIP results are obtained in the form of raw data representing cumulative intruded volume versus pore diameter curves and logarithmic differential intruded volume versus pore diameter of cement mortar curves.

## 3. Pore Size Distribution of Cement Mortar

### 3.1. Test Results

#### 3.1.1. Manufactured Sand Mortar

**Figure 2:**
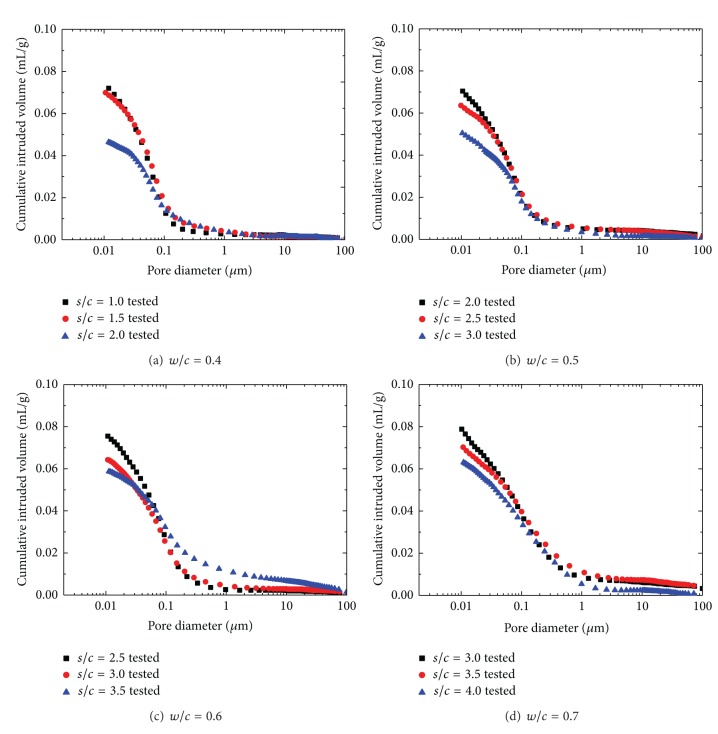
Cumulative intruded pore volume versus pore diameter for MS mortars.

**Figure 3:**
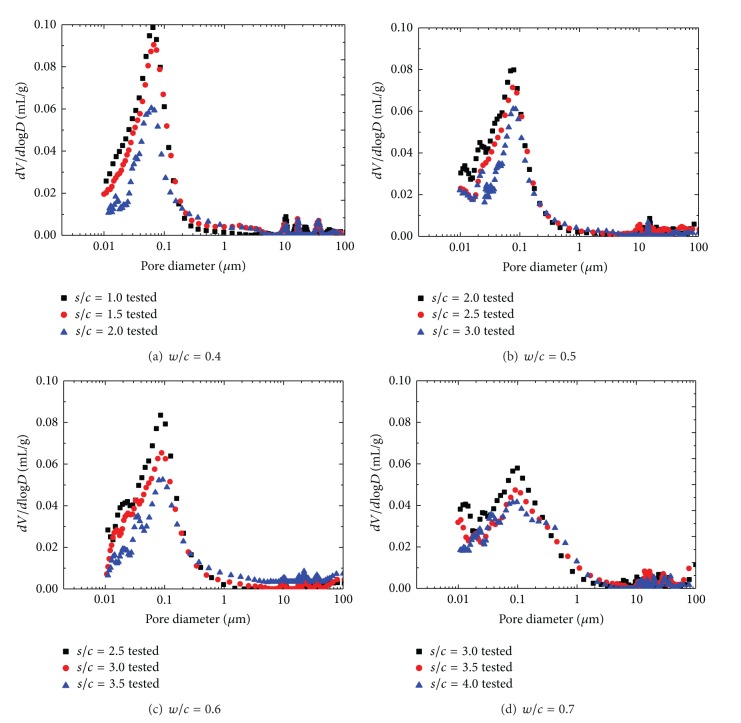
Differential intrudes volume versus pore diameter for MS mortars.

In this section, the pore structure of manufactured sand mortars with different mix proportions after 28 days of curing is discussed. The cumulative intrusion versus pore diameter curves and differential curves is shown in Figures 2 and 3, respectively. And detailed pore structure parameters are listed in Table 3.

The MIP test data indicate a threshold radius below which there is relatively little intrusion and immediately above where rapid intrusion commences. This corresponds to the region of inflection, following an almost horizontal portion of cumulative intrusion curves. The test results also indicate that the threshold radius increases with increasing fine aggregate and water to cement ratio. If the threshold radius is assumed to be the initial intergranular spacing at the setting time, the higher water to cement ratio generates a higher threshold radius. As aggregate volume concentration increases, the threshold region tends to flatten out and the threshold radius increases (228 nm–1015 nm) progressively, which can be attributed to the fine aggregate effect of the reorientation of the pore system of mortar. Different from cement paste, the threshold radius in the mortar is linked to the binder-aggregate interface or even fissures rather than to the pores alone [26]. Feldman [27] demonstrated that the total intruded pore volume decreased with increasing aggregate volume concentration. However, intruded pore volume per volume of paste in the mortar increases with increasing aggregate volume concentration. This strongly suggested that pores being intruded by mercury may not be pores in the paste alone but could include fissures and bond cracks at the aggregate-paste interface.

It can be found in Figure 3 that the character of pore size distribution curves of manufactured sand mortars is influenced by both *w*/*c* and *s*/*c*. The difference in pore structure between cement mortar of different *w*/*c* lies mainly in the region of large pores; mortar with higher *w*/*c* has a group of larger pores. This is consistent with experimental research from Gonçalves et al. [28]. Differential curves for mortars with lower *w*/*c* exhibit a sharply defined initial peak indicating a unimodal distribution of pore sizes. As *w*/*c* increases, a second more rounded peak appears at a larger pore size, thus presenting a bimodal distribution. The presence of a sharply defined intrusion peak in the differential curve indicates the intrusion of mercury throughout a pore network connected to the specimen surface [29, 30]. Therefore, the initial intrusion peak observed here corresponds to the minimum throat dimension of an interconnected capillary network. The rounded peak appears in the pore size of above 10 mm, which is probably favored in large aggregate volume concentration and *w*/*c*. The most probable pore size corresponding to initial peak ranges from 65 nm to 100 nm in MS mortars and increases with both the increment of fine aggregate and *w*/*c* ratio.

#### 3.1.2. Natural Sand Mortar

**Figure 4:**
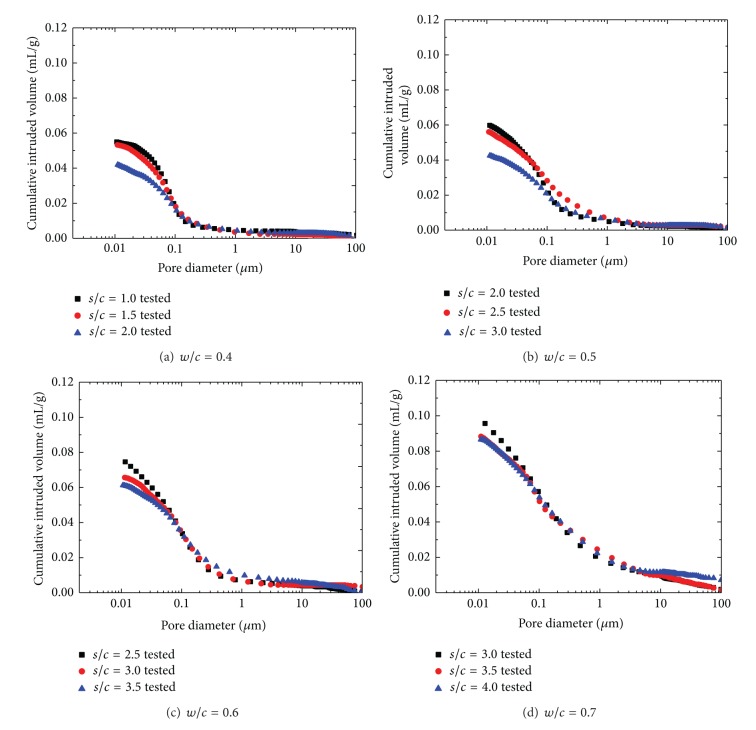
Cumulative intruded pore volume versus pore diameter for NS mortars.

**Figure 5:**
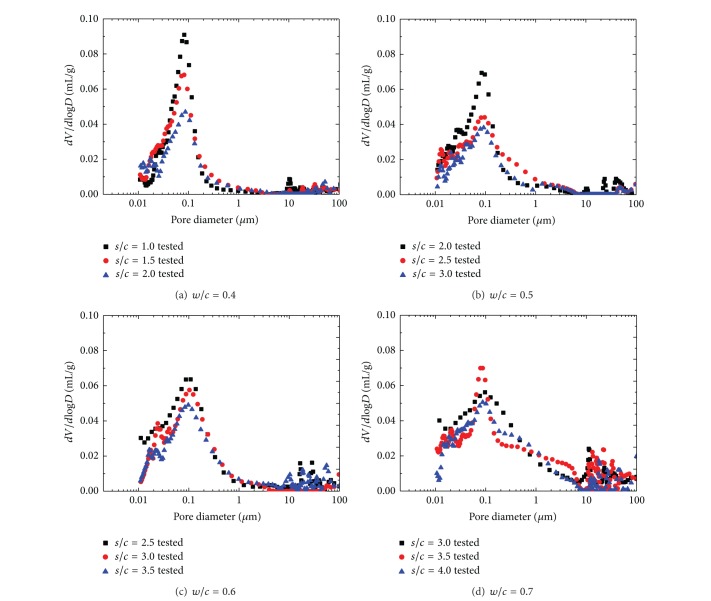
Differential intrudes volume versus pore diameter for NS mortars.

The cumulative intrusion versus pore diameter curves and differential curves of natural sand mortar is shown in Figures 4 and 5, respectively. And detailed pore structure parameters are listed in Table 4.

It can be found from the test results that the influence of *w*/*c* and *s*/*c* on characteristics of the pore structure of the natural sand mortar is similar to the manufactured sand mortar. The total porosity of the manufactured sand mortar is higher, which is ascribed to the angular shape and rough texture of manufactured sand [2, 31]. However, due to its better particle size distribution, the manufactured sand mortar has finer threshold radius and probable pore size natural sand mortar.

### 3.2. Comparison Test Results with Existing Models

An appropriate pore size distribution model of cement-based materials determined by MIP should be one that not only fits the experimental data but also has the reasonable physical meaning of pore structure. Historically, several general types of models have been proposed for pore size distribution of cement-based materials [18–22], as shown in Table 4.

**Figure 6:**
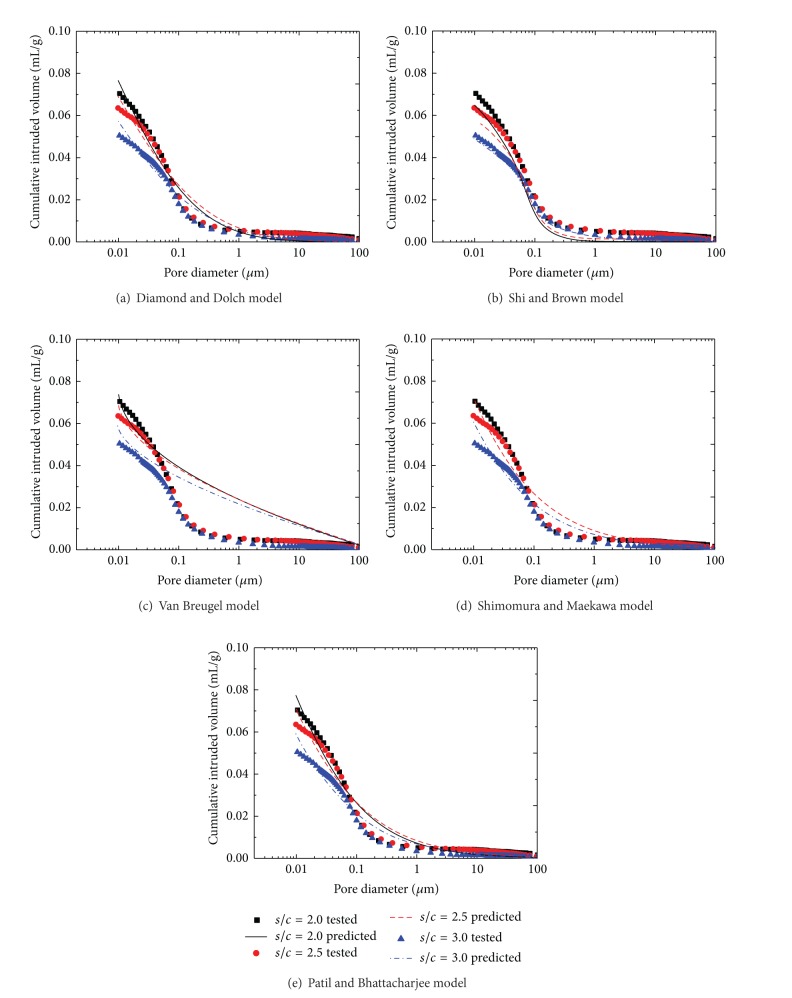
Comparison between test and predicted results by existing models for manufactured sand mortar.

**Figure 7:**
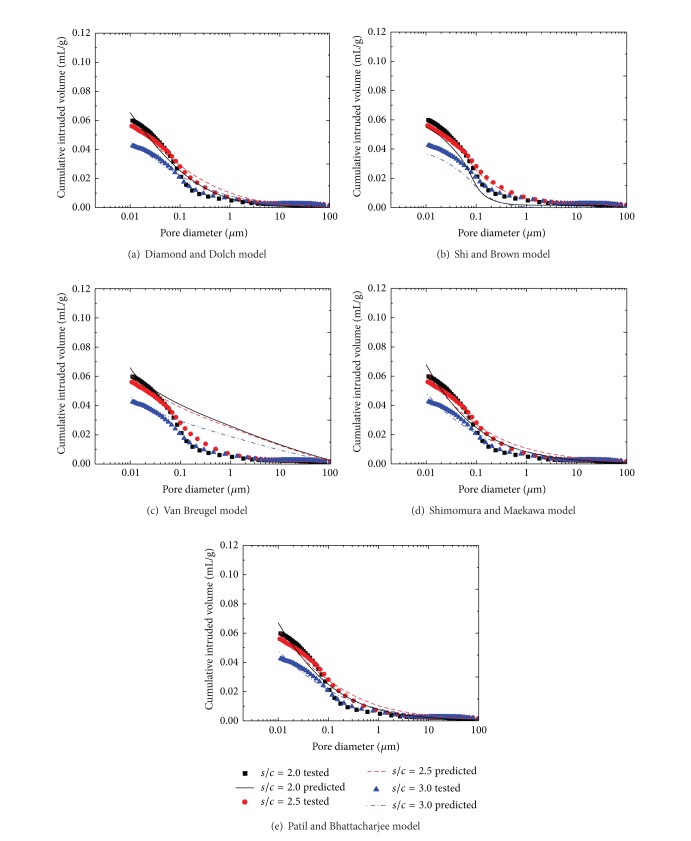
Comparison between test and predicted results by existing models for natural sand mortar.

In this experimental research, the fit of existing models of distribution functions was examined for three mix proportions at *w*/*c* = 0.5 cement mortars of varying *s*/*c* prepared with manufactured sand and natural sand as the representatives, as shown in Figures 6 and 7, respectively.

By comparison to experiments, as shown in Figures 6(a) and 7(a), if both fine and coarse pores are included in cement mortar, a single lognormal distribution model is not adequate. Figures 6(c) and 7(c) show that the Van Breugel model has poor precision. The simulated distribution is coarser than those obtained by the MIP test when the pore size is coarser than 0.1 mm, which is the simulation overestimating the volume of larger pores. This may be related to the real world flocculation of cement particles, which is not included in the Van Breugel model.

Compound lognormal distribution is excellent to simulate the pore size distribution by MIP test, as shown in Figures 6(b) and 7(b); 2 nm to about 0.2 mm have been included in this compound lognormal model. The first subdistribution is associated with coarse pores that may extend to voids. The third subdistribution is associated with fine pores that may extend to gel pores. The middle distribution includes capillary pores. Shimomura and Maekawa and Patil and Bhattacharjee models have both directly given the relationship between the cumulative intruded volume and pore diameter. The simulated results and experimental data by MIP showed a good correlation.

## 4. Strength-Pore Structure Relationship

### 4.1. Porosity

**Figure 8:**
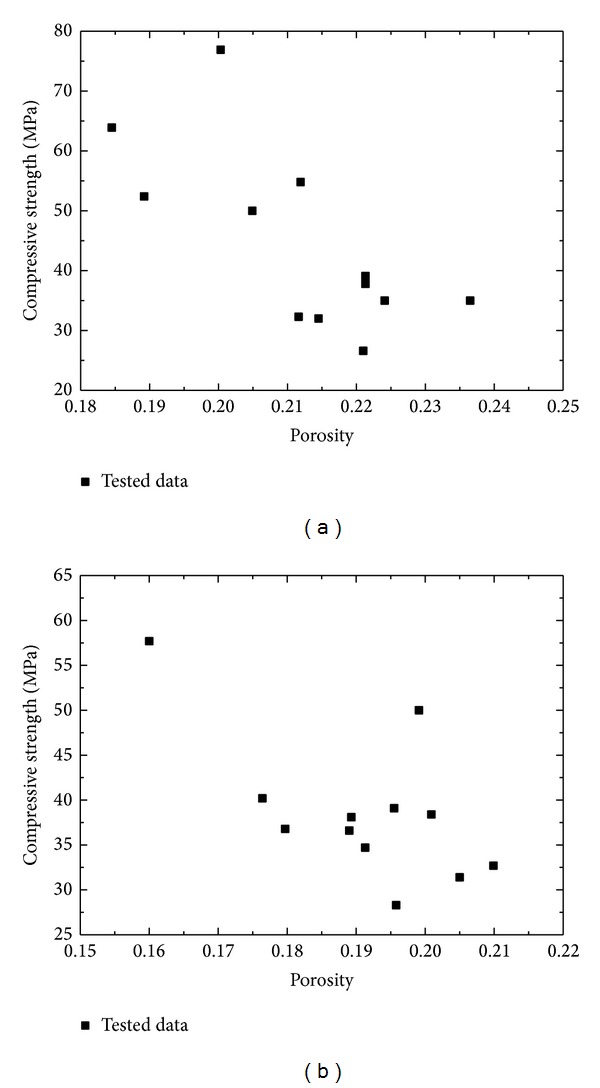
Compressive strength versus porosity of cement mortar: (a) manufactured sand and (b) natural sand.

Taking an empirical approach, connections between the porosity and strength of solid materials have been established by many researchers [8–11]. Based on these models, the relationship between strength and porosity of cement mortar had been given by Chen et al.  [12]; meanwhile, a model to predict strength was proposed. The simulated results have shown that the extended Zheng's model is a good representation of the experimental data on the strength of cement mortar. In this paper, experimental results show that strength and porosity have obvious discreteness, as shown in Figure 8, which results in the existing models being unavailable to simulate the relationship between strength and porosity of cement mortar.

### 4.2. Pore Structure

In traditional researches, porosity is the only influential factor on the strength of cement-based materials taken into consideration, which is empirical or semiempirical. This experimental data results in these existing models being unavailable to simulate the relationship between strength and porosity of cement mortar. Therefore, other pore structure parameters such as mean diameter should be taken into account. Cumulative intruded volume versus pore diameter curve is divided into *n* shares averagely and *V*_*i*_ represents the volume of pore diameter *d*_*i*_. The mean diameter can be calculated as follows:

(2) $$\begin{array}{c} \ln d_{m}=\frac{\sum_{i=1}^{i=n}V_{i}\ln d_{i}}{\sum_{i=1}^{i=n}V_{i}}, \end{array}$$

where *d*_*m*_ is the mean diameter and *d*_*i*_ is the *i*th pore diameter.

The mean diameter *d*_*m*_ is considered to account for the influence of pore structure on the compressive strength of concrete:

(3) $$\begin{array}{c} \sigma =KC\frac{\left(1-P\right)}{\sqrt{d_{m}}}, \end{array}$$

where *d*_*m*_ is mean diameter, *P* is the total porosity, *C* is cement content, and *K* is parameter.

Kondraivendhan and Bhattacharjee [32] have proposed a relationship between compressive strength and hydration degree, mean diameter, and porosity as follows:

(4) $$\begin{array}{c} \sigma =KC\alpha \frac{\left(1-P\right)}{\sqrt{d_{m}}}, \end{array}$$

where *K* is empirical parameter, *C* is cement content, and *α* is hydration degree.

In this paper, an extended Bhattacharjee model is proposed as follows:

(5) $$\begin{array}{c} \sigma =K\exp\left(B\alpha \frac{\left(1-P\right)}{\sqrt{d_{m}}}\right), \end{array}$$

where *K* and *B* are experimental parameters.

**Figure 9:**
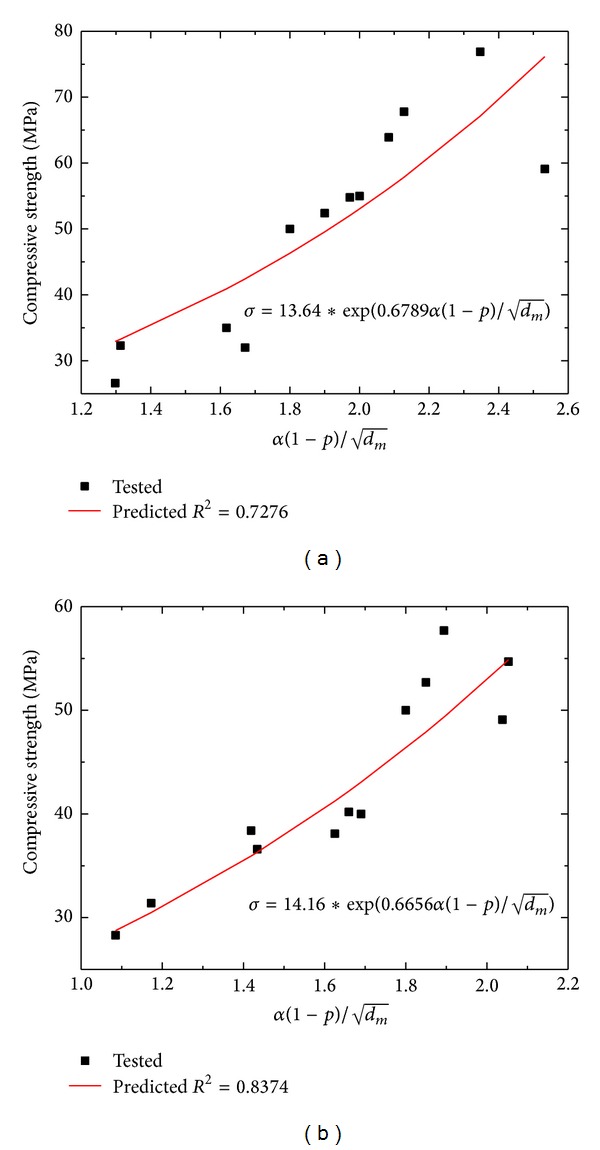
Relationship between experimental results of compressive strength and model formula $\alpha (1-p)/\sqrt{d_{m}}$: (a) manufactured sand mortar and (b) natural sand mortar.

Figure 9 shows the relationship between compressive strength and $\alpha (1-P)/\sqrt{d_{m}}$. Porosity, mean diameter, and hydration degree are taken into consideration, which clearly explain the relationship between pore structure and strength of cement mortar.

## 5. Conclusions

1. Porosity of manufactured sand mortar is higher than that of natural sand mortar, and the compressive strength is higher than that of natural sand mortar, which demonstrates that porosity is not the only influential factor on compressive strength of mortar. The probable pore size and threshold radius of manufactured sand mortar are finer. Besides, the probable pore size and threshold radius increase with increasing water to cement ratio and sand to cement ratio.
2. The existing models of pore size distribution of cement-based materials have been reviewed and compared with test results in this investigation. A single lognormal distribution may not be adequate if both fine and coarse pores are included in cement mortar. Although too many parameters are included in the model, the compound lognormal distribution is excellent to simulate the experimental pore size distributions. The Van Breugel model has poor precision. Shimomura and Patil models are similar, and the parameter is simple and particular with *w*/*c* and *s*/*c* and so on.
3. The extended Bhattacharjee model was built to examine the relationship between compressive strength and pore structure. Porosity, mean diameter, and hydration degree were taken into consideration, which clearly explain the relationship between pore structure and strength of cement mortar.

## Figures and Tables

**Table 1:** Summary of the characteristics of the fine aggregate samples tested.

Sample	Specific gravity	Bulk density (g/cm^3^)	Void (%)	Fineness modules
MS	2.63	1.72	35.86	2.4
NS	2.53	1.65	34.78	2.6

**Table 2:** Mix proportion and workability of mortar.

Manufactured sand				Natural sand			
No.	*W/C *	*S/C *	Fluidity (mm)	No.	*W/C *	*S/C *	Fluidity (mm)
M1	0.4	1.0	257.0	N1	0.4	1.0	267.5
M2	0.4	1.5	215.5	N2	0.4	1.5	250.0
M3	0.4	2.0	162.0	N3	0.4	2.0	197.5
M4	0.5	2.0	258.0	N4	0.5	2.0	262.5
M5	0.5	2.5	187.0	N5	0.5	2.5	227.5
M6	0.5	3.0	150.5	N6	0.5	3.0	180.0
M7	0.6	2.5	265.5	N7	0.6	2.5	280.0
M8	0.6	3.0	237.0	N8	0.6	3.0	250.0
M9	0.6	3.5	150.5	N9	0.6	3.5	212.5
M10	0.7	3.0	267.0	N10	0.7	3.0	287.5
M11	0.7	3.5	242.0	N11	0.7	3.5	265.0
M12	0.7	4.0	178.0	N12	0.7	4.0	235.0

**Table 3:** Parameters of pore structure of cement mortar.

No.	Porosity (%)	Threshold radius (nm)	Probable pore size (nm)	No.	Porosity (%)	Threshold radius (nm)	Probable pore size (nm)
M1	22.30	202.3	66.7	N1	20.99	228.1	82.29
M2	20.03	246.2	67.2	N2	19.13	242.9	79.56
M3	18.48	186.5	65.8	N3	16.00	242.9	90.07
M4	22.23	216.5	77.0	N4	19.55	561.8	90.07
M5	21.30	220.9	78.7	N5	17.97	1089.0	88.75
M6	19.09	246.2	81.1	N6	17.64	561.8	92.32
M7	22.53	150.1	86.1	N7	19.91	562.1	103.2
M8	20.51	160.0	89.8	N8	18.93	562.1	107.8
M9	21.26	182.7	92.8	N9	18.90	562.1	102.0
M10	23.87	491.5	95.2	N10	20.09	959.1	97.0
M11	22.22	504.3	96.5	N11	20.50	959.1	87.11
M12	21.60	574.4	98.2	N12	19.58	1015.0	93.8

**Table 4:** Pore size distribution models of cement-based materials.

Models	Equations	Parameters
Diamond and Dolch model [18]	$f\left(d\right)=\frac{1}{\sqrt{2\pi }\ln\sigma }\exp\left[-{\left(\frac{\ln\left(d^{*}\right)/{\overset{-}{d}}^{*}}{\sqrt{2}\ln\sigma }\right)}^{2}\right]$	${\bar{d}}^{*},\sigma$

Van Breugel model [19]	$V_{\left(\leq d\right)}=a\cdot {\left[\ln\left(\frac{d}{d_{0}}\right)\right]}^{n}$	*a*, *n*

Shi and Brown model [20]	$p\left(x\right)=\frac{1}{\sqrt{2\pi \sigma^{2}x}}\exp\left[-\frac{1}{2}{\left(\frac{\log\left(x\right)-\mu }{2}\right)}^{2}\right]$ *p*(*x*) = ∑*f*_*i*_ *p*(*x*, *μ*_*i*_, *σ*_*i*_), ∑*f*_*i*_ = 1	*μ*_*i*_, *σ*_*i*_

Shimomurat and Maekawa model [21]	*V*_(*d*)_ = *V*_0_[1 − exp⁡(−*Bd* ^*C*^)]	*B*, *C*

Patil and Bhattacharjee model [22]	$V=\frac{\phi d_{0.5}^{m}}{d^{m}+d_{0.5}^{m}}$	*r*_0.5_, *m*
